# Progress in the efficacy and mechanism of spinal cord stimulation in neuropathological pain

**DOI:** 10.1002/ibra.12020

**Published:** 2022-03-02

**Authors:** Shun‐Lian Li, Jing Li, Hui‐Chan Xu, Yu‐Cong Liu, Ting‐Ting Yang, Hao Yuan

**Affiliations:** ^1^ Department of Anesthesia Zunyi Medical University Zunyi Guizhou China; ^2^ School of Basic Medicine Kunming Medical University Kunming Yunnan China; ^3^ Department of Spine Surgery Affiliated Hospital of Zunyi Medical University Zunyi Guizhou China

**Keywords:** efficacy, neuropathological pain, progress of mechanism, spinal cord stimulation

## Abstract

Neuropathic pain (NP) is a long‐term recurrent disease caused by somatosensory nervous system injury, with spontaneous pain, hyperalgesia, ectopic pain, and paresthesia as the main clinical manifestations. It adversely affects patients' quality of life. NP treatments often include medication, physical therapy, and invasive therapy; the first two therapies are generally ineffective for some NP patients. These patients sometimes rely on invasive therapy to alleviate pain. Spinal cord stimulation (SCS) is a very effective therapeutic method. SCS is a neuroregulatory method that involves placing the electrodes on the corresponding painful spinal cords. Pain is greatly alleviated after SCS. SCS has been proven to be an effective therapeutic method for the treatment of neurological pain. Furthermore, SCS provides a feasible approach for patients with unsuccessful drug treatment. This paper reviews the relevant literature of spinal cord electrical stimulation, focusing on the mechanism of action, clinical application, clinical efficacy and technical progress of spinal cord electrical stimulation. SCS is widely used in the treatment of NP diseases such as postherpetic neuralgia, back surgery failure syndrome, and phantom limb pain. With advancements in science and technology, tremendous progress has also been made in the spinal cord electrical stimulation method and good momentum has been maintained.

## Graphical abstract

The components of the image are redirected to tell the story from top to bottom. For the sake of simplicity, I have used the two most representative diagrams to illustrate my review. The review focuses on the latest mechanisms and applications of SCS.

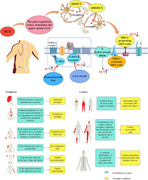

## 1 INTRODUCTION

Neuropathic pain (NP) is a common chronic pain condition caused by pathological changes or diseases of the somatosensory nervous system, which places a huge burden on people's life.^1^ Neuropathy can directly damage the somatosensory system. It is indirectly elicited by metabolic stress changes, autoimmunity or inflammation of nerves or their peripheral areas, and so forth.^2^, ^3^ Generally speaking, the effectiveness of medication is usually limited. Fortunately, spinal cord stimulation (SCS) can be used to treat a wide range of NP including postherpetic neuralgia (PHN), failed back surgery syndrome (FBSS), phantom limb pain, diabetic neuropathy, cephalic and facial neuralgia, genital neuralgia, and so forth.^4^ SCS involves the use of electrical impulses for signal stimulation of spinal cord nerves to treat disease. By inserting a pulse mode spinal cord stimulator, there can be effective improvements in patients' function and significant pain relief can also be achieved.^5^ This paper focuses on the curative effect and mechanism of SCS in the treatment of NP. Based on our observations, it was found that it can inhibit or alleviate pain by blocking pain signal transmission, interfere with the pain pathway, activate the opioid pathway,^6^ stimulate the locus coeruleus system, and regulate γ‐aminobutyric acid (GABA). Meanwhile, there have been advances in the SCS method in recent years. High‐frequency spinal cord stimulation (HF‐SCS) and dorsal root ganglion stimulation (DRGS)^7^ are characterized by good efficacy and few adverse reactions.

## 2 NP

The International Association for the Study of Pain defines NP as pain caused by injury or disease of the somatosensory nervous system,^8^ including peripheral nerve fibers (Aβ, Aδ, and C fibers) and central neurons, and 7%–10% of the general population suffers from this disease.^9^ It is a challenge to treat chronic pain syndromes clinically. Increasingly more data show that opioid medication is inappropriate and fails to relieve pain, especially chronic and long‐term pain, and affects patients physiologically and psychologically.^10^ Its interference with the steady internal environment triggers metabolic disorders, arrhythmia, cardiopulmonary insufficiency, stress ulcers, and other complications.^11^ As neuropathological pain mostly manifests as chronic pain, pressing pain, spontaneous pain, pain allergy, hypersensitivity, and other clinical manifestations that severely disrupt daily life, neuropathological pain should receive due attention.^12^ Treatment can be found by studying the mechanism, development, and maintenance of NP.^13^ Somatic nervous system injury or disease in the peripheral or central nervous areas leads to the production of NP in humans. Peripheral NP is more common than central NP and is considered to be caused by peripheral mechanisms after nerve injury^14^ (Table 1).

**Table 1:** Major types and research models of neuropathological pain^15^

Classification	Disease	Study model
Peripheral neuralgia	Postoperative neuralgia, posttraumatic neuralgia, pain phantom limb, neuroneuralgia after neuralgia, pain‐induced diabetic neuropathy, complex regional pain syndrome, neuroroot neuropathy, malstrophic neuropathy, HIV neuropathy	Neuroma model, chronic compression injury (CCI) model, facial pain model
		Diabetic neuropathic pain model, postherpetic neuralgia model, trigeminal neuralgia model
Central neuralgia	Spinal cord injury pain Spinal cord deficiency pain, Parkinson's disease pain Multiple sclerosis disease pain	Toxic spinal cord injury, Photochemical damage model, Weight loss or contusion, spinal cord injury

### 2.1 Activation of astrocytes in the dorsal spinal cord (central sensitization)

**Figure 1:**
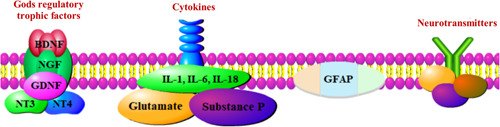
Activation of astrocytes in the dorsal spinal cord. BDNF, brain‐derived neuropathic factor; GDNF, glial‐derived neurotrophic factor; GFAP, glial fibrillary acidic protein; NGF, nerve growth factor; NT3, neurotrophic factor 3; NT4, neurotrophic factor 4 [Color figure can be viewed at wileyonlinelibrary.com]

The mechanism of pain has been studied mainly through neurons, but glial cells, the most widely distributed and abundant cells in the central nervous system, play a major role in pain. NP is a pathological condition that results from structural and functional changes that occur after nerve damage.^16^ A wide range of functional, structural, and molecular changes in the glia consistent with neuronal changes were found in an animal model of neuronal damage of NP.^14^ Recent studies of astrocytes have all shown that glial cells are crucial to these changes and play an important role in the development and maintenance of neuropathological pain.^17^ Neurotransmitters released by astrocytes, regulatory nutritional factors (such as brain‐derived neuropathic factor [BDNF]), cytokines (glutamate, substance P, interleukin [IL]‐1, IL‐6, IL‐18,^18^ Shenzhu growth factor, etc.), are all involved in the transduction and regulation of pain signals.^19^ Related studies have shown that IL‐18 and the IL‐18 receptor (IL‐18R) are induced in the spinal dorsal horn. After nerve injury, the expression of IL‐18 and IL‐18R in the dorsal horn is significantly increased, and microglia overactivity and astrocytes are upregulated, respectively. Injury‐induced tactile pain is mitigated by inhibition of the IL‐18 signaling pathway, which reduces phosphorylation of nuclear factor κB and induction of astrocyte markers in spinal astrocytes. Therefore, blocking the IL‐18 signaling pathway in glial cells may provide an effective strategy for the treatment of NP, which may play an important role in allotactile pain after nerve injury.^18^ Other research has studied glial fibrillary acidic protein (GFAP) as a specific marker of spinal astrocytes that is often used to detect the expression of an astrocyte, and it has been found that Schwann cells in particular have unique regenerative properties that can repair pudendal neuralgias (PNS) after injury, making them almost impossible to find similar second cells in our bodies,^20^ which has important implications for distinguishing Schwann cells in developing nerves.^21^ Myelinated axons are nourished by the production of various growth factors such as nerve growth factor (NGF), glial derived neurotrophic factor (GDNF), neurotrophic factor 3 (NT3), and neurotrophic factor 4 (NT4) on long axons^22^ (Figure 1).

### 2.2 C‐fibers are involved in the mechanism of neuropathological pain

Initially, C‐fibers were considered to be simple nerve fibers that transmit information about action potentials; pain occurs when primary afferent C fibers are activated by harmful stimuli, but C fibers may be involved in more complex neural processes than the simple one‐to‐one relationship between sensory and receptor types, based on the marker line encoding (the key encoding mechanism for stimuli).^23^ In other words, the conduction coding function of C‐fiber can cause information to change in frequency or pattern during the conduction of action potential, the performance is that under the same external intensity stimulation, the action potential of afferent nerve increases, the release of transmitter increases, and the excitability increases, which is one of the possible mechanisms leading to peripheral sensitization and central sensitization.^24^ When peripheral receptors are damaged and stimulated, the introduction of signals into the C‐fiber increases the stimulus‐dependent excitability of neurons in the spinal dorsal horn; this is called central sensitization. Long‐term enhancement between C‐fibers and interneursynapses is the basis of central sensitization and neuropathological pain.^25^ Continuous peripheral stimulation increases peripheral nerve sensitivity, and causes hyperalgesia and touch‐induced pain.^26^ Spontaneous pain, hyperalgesia, numbness, burning, coldness, and other symptoms of limbs in diabetic patients are mainly attributed to the functional changes of unmyelinated C‐fibers.^27^ Low‐intensity electrical stimulation of the sciatic nerve in L5~6 spinal nerve ligated rats induced LTP in C‐fiber‐evoked potential, while the same intensity of stimulation did not induce LTP in normal rats, suggesting that sensitivity to pain was related to the regulation of C‐fiber sensitization. Relevant studies have also found that two abnormal properties developed in undamaged skin C‐fiber injury receptors on spinal nerve ligation: spontaneous activity and adrenergic sensitivity. Abnormal function of these pain receptors may lead to neurological pain.^28^

### 2.3 The downlink suppression system function is decreased

The downlink inhibitory system is a regulatory system that transmits peripheral damaging signals to the center, activates central inhibitory neurons, and reduces the pain response. The descending pathway regulating damaging signaling originates from the gray, blue spots, anterior buckback, amygdala, and the hypothalamus, and through the aqueducts around the gray matter and medulla to the spinal cord.^29^ Transmitters involved in pain downside inhibition regulation include norepinephrine, 5‐HT, dopamine, and endogenous opiates.^9^, ^29^ Neuronal dysfunction in NP patients causes an imbalance between downside depression and excitability, leading to pain, anxiety, depression, and sleep problems.^9^ One of the core mechanisms of the occurrence and development of chronic pain may be the dysfunction of the pain downregulation system.^30^ Related studies have shown that spinal cord progenitor cells seem to be activated by SCS through a descending pathway, thereby alleviating NP.^31^

### 2.4 Release of tumor necrosis factor (TNF)‐α

TNF is a small‐molecule protein produced by phagocytosis of bacterial infection or other immune sources. Aggarwal et al.^32^ were the first to define TNF as two distinct types based on structure and source, namely, TNF‐α and lymphotoxin or TNF‐β. It is mainly produced by mono‐macrophages and not only causes tumor cell death but also plays a role in the pathogenesis of pain, including nerve pain, inflammatory pain, and cancerous pain.^33^ When peripheral nerve injury occurs, instantaneous upregulated TNF‐α can activate proinflammatory media to produce waterfall responses.^34^ Under conditions of neurological pain, axons can also damage and regulate the activity and sensitivity of damaging receptors^35^; meanwhile, TNF‐α also plays a core role in central sensitization, and TNF‐α can promote central sensitization by increasing glutamate release in the presynaptic membrane and increasing *N*‐methyl‐d‐aspartate receptor (*N*‐methyl‐d‐aspartic acid receptor, NMDA‐R) activity, or by increasing synaptic transmission and excessive excitability of spinal dorsal horn neurons.

## 3 SCS MECHANISMS FOR NEUROPATHOLOGICAL PAIN

**Figure 2:**
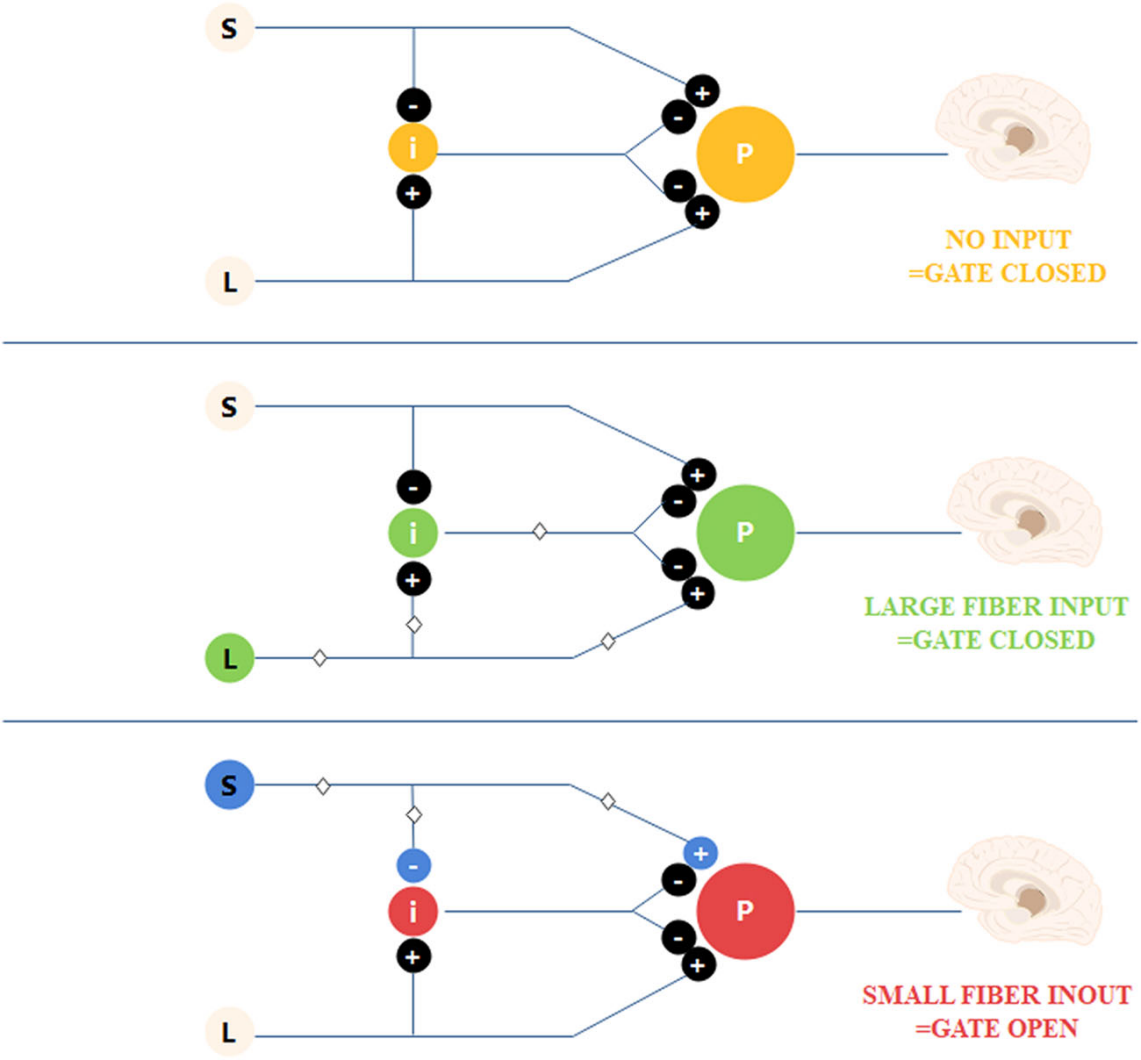
The Melzack–Wall pain gate. Gate mechanism: without input, the gate is closed; the coarse fiber impulse leads to closure of the gate; and the fine fiber impulse induces opening of the gate [Color figure can be viewed at wileyonlinelibrary.com]

Since 1967, people have treated multiple chronic pain disorders using SCS.^36^ SCS is an adjustable, nondestructive neuromodulation that can use therapeutic doses of electricity to treat NP.^36^ SCS can be used for the treatment of various kinds of pain, such as neurogenic pain, including postlaminectomy syndrome, complex regional pain syndrome, spinal cord injury pain, and interstitial cystitis. Indications also include intractable pain and neurogenic thoracic exit syndrome caused by abdominal or visceral pain.^4^ SCS has been successfully used in the treatment of severe pain caused by lower limb ischemic disease and refractory pain. Studies have shown that SCS reduces pain by more than 50% in 50%–60% of voluntary patients. Melzack and Wall^37^ published the gating theory in 1965. It lays the theoretical foundation for SCS, arguing that the “electric‐chemical” information of peripheral pain is transmitted into the spinal cord through thin unmyelinated C‐fibers and a small amount of myelinated A‐δ fibers that terminate in the spinal cord, and the “door” receiving the fiber information, which can retrograde‐suppress the pain information of fiber transmission.^37^ Conduction of both coarse fibers (Aa, Aβ) and fine fibers (Aδ, C) activates T cells in the posterior corner of the spinal cord while simultaneously forming a synaptic association with the SG cells in the posterior angle. Coarse nerve fiber excitation activates T cells and SG cells, closing the gate to prevent impulse transmission and thus attenuating or eliminating pain sensation. Fine fiber excitation inhibit SG cells only, so that the gate opens to produce pain^37^ (Figure 2). However, the gate theory cannot fully explain the analgesic mechanism of SCS.^38^ Currently, there is no complete theoretical explanation of the analgesia mechanism of SCS, but relevant studies show that the treatment of SCS may also have the following mechanism.

### 3.1 Interference with pain pathways

A dorsal column–brainstem–spinal cord loop was found in the animal model, suggesting that implanted electrodes in the spinal cord may reduce the conduction or remodeling of incoming sensation of pain in the spinal thalamus bundle and interrupt the central perception of pain,^39^ stimulate neurons above the spinal cord, and affect the conduction or regulation of pain sensation. Neuroimaging studies have shown that tonic SCS induces analgesia mainly by regulating the lateral pain upstream pathway and by interfering with electrical and metabolic activities in the cingulate gyrus, lateral sensory thalamic nucleus, prefrontal cortex, and posterior central gyrus.^39^, ^40^ Neuroimaging studies^41^ have also shown that SCS mainly functions by regulating pain upstream pathways, interfering with the metabolic activity of the buckle back, lateral sensory thalamic nucleus, prefrontal cortex, and central posterior return.

### 3.2 Activation of the opioid channel

Increasingly more evidence suggests that δ opioid receptors are attractive therapeutic targets for various forms of chronic pain and certain mood disorders, including depression and anxiety.^42^ Also, δ agonists are effective in preclinical models of chronic pain, including those used for NP, inflammatory pain, and cancer.^43^ Combined administration of LY 218324, an endocannabinoid reuptake/breakdown inhibitor, partially reverses early SCS sensitivity to pain. Moreover, the amplification was inhibited by the CB1 R antagonist AM251, but not by the CB2 R antagonist AM630. The use of naloxone reduced the reversal of pain allergy induced by early SCS, indicating the important role of opioids in SCS.^44^ Sato et al.^6^ found that antagonizing different opioid receptors could inhibit the analgesic effects of different frequencies of SCS, suggesting that the opioid receptor was associated with the analgesic mechanism of SCS, suggesting that 4 Hz SCS induced analgesia by activation of μ‐receptors and 60 Hz SCS produced analgesic effects by activation of the δ‐receptor.

### 3.3 Stimulation of the locus coeruleus system

In the brain structure that receives the papillogenesis projection of histaminergic tubercles, the bridging blue spot (LC) is involved in antihypertensive norepinenergic control of pain. The use of zolentine or pyridine alone in LC does not affect pain behavior, while A‐960656 (histamine H3 receptor antagonist) inhibits hypersensitivity. A reasonable explanation for these findings is that neuropathy hypersensitivity is promoted due to the histamine H2 receptor mediated by histamine on the norepinephrine receptor in α1/2‐adrenergic cells. Blocking of the self‐inhibitory histamine H3 receptors at the end of the histaminergic nerve in LC facilitates the release of histamine, thereby increasing the pain inhibition of antihypertensive norepinenergic activity.^45^ Song et al.^46^ found, in the rat model, that SCS passes the gray‐mass area around the midbrain duct, the ventromedial region of the extended medullary head (rostral ventromedial medulla, RVM). Control of downward pain is achieved by stimulating RVM. SCS increases the release of injury‐resistant OFF cells and serotontamine‐like cells in RVM without affecting injury‐promoting OFF cells in RVM, producing analgesic effects by activating these downlink inhibitory signals that start or relay to RVM.

### 3.4 Regulation of the γ‐aminobutyric acidergic (GABAergic) system

**Figure 3:**
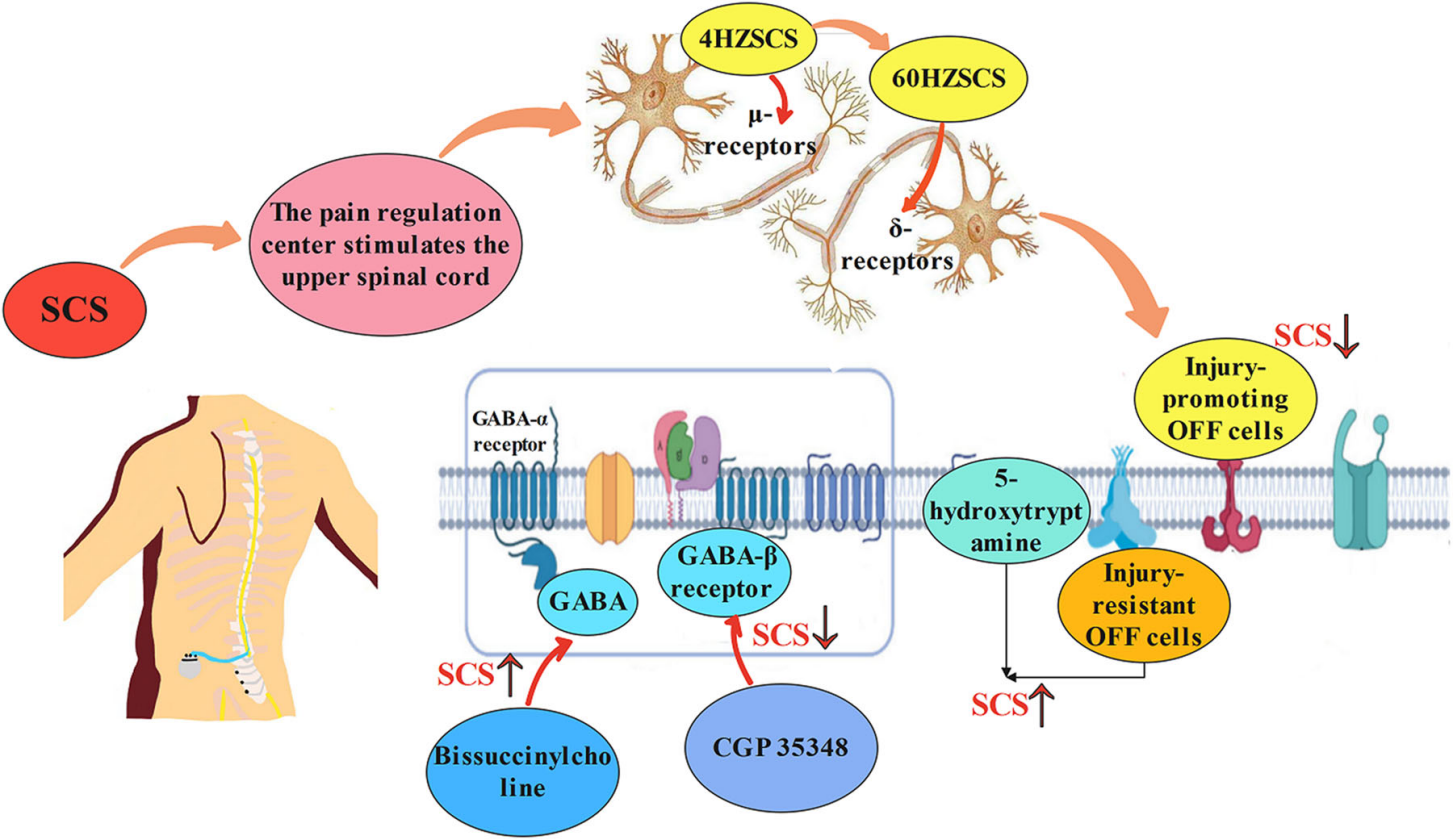
Spinal cord stimulation (SCS) mechanisms for neuropathological pain. SCS exerts analgesic effects by acting on pain regulation centers to stimulate the upper spinal cord. The SCS passes 40 Hz SCS produce analgesia by activating μ‐receptors, while 60 Hz SCS produce analgesia by activating δ‐receptor. SCS achieves analgesic effects by stimulating the blue spot system and GABAergic. GABA, γ‐aminobutyric acid [Color figure can be viewed at wileyonlinelibrary.com]

The wide‐dynamic range (WDR) neuronal contraction input caused by excessive pain triggers the lateral pain pathway, allowing abnormal conduction of pain sensation to reach the brain. SCS‐increased GABA release neutralizes the hyperexcitability of dorsal horn large dynamic range (WDR) neurons.^47^ Relevant studies have shown the effect of the loss of inhibition on the SCS action due to the loss of GABA or KCC2 function. By weakening the input of GABA energy‐intermediate neurons or connection to the WDR neurons and the anion reverse potential of moving WDR neurons upward, one of the effects of local and surrounding GABA energy‐intermediate neurons is reduced, thus reducing the range of optimal SCS frequency and changing the frequency at which SCS had a maximal effect. It has been reported that GABA is related to SCS.^48^ Related studies have found that tonic SCS induces GABA release from inhibitory intermediate neurons in the spinal dorsal horn mainly through a segmental spinal mechanism.^49^ Zhang et al.^50^ found that the GABAergic mechanism can regulate SCS‐mediated neuronal responses, and the application of the GABA‐α receptor antagonist bissuccinylcholine can stimulate SCS‐mediated excitability and exert an analgesic effect, while the application of the GABA‐β receptor antagonist CGP35348 can inhibit the analgesic effect of SCS. Presumably, the SCS controls the sensory neuronal projection activity and neuronal response mediated by SCS by regulating the GABAergic system (Figure 3).

## 4 CLINICAL APPLICATION OF SCS IN TREATING NEUROPATHOLOGICAL PAIN

**Figure 4:**
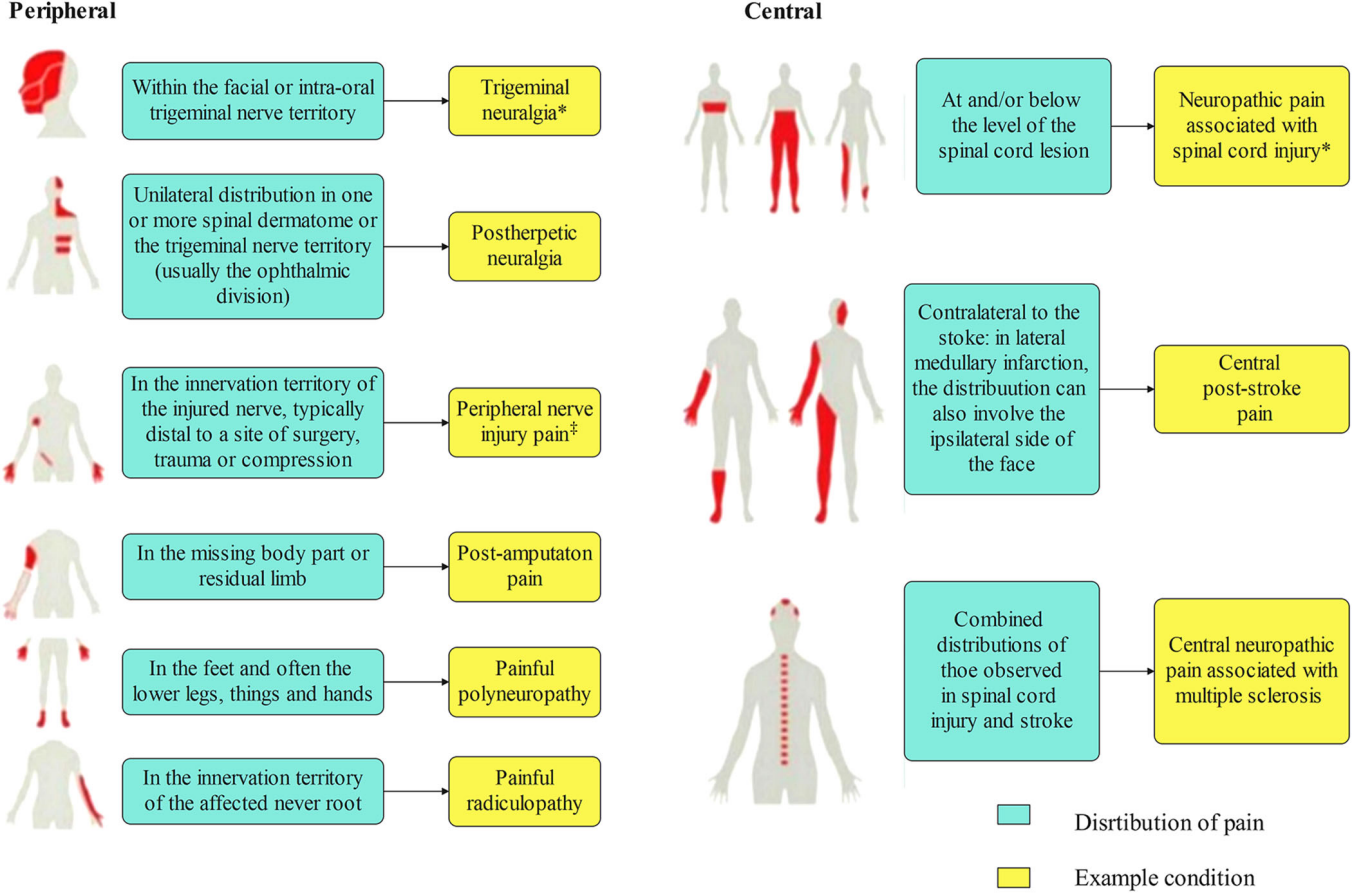
Neuroanatomical distribution of pain symptoms and sensory signs under neuropathic pain.  Peripheral and central distribution of pain and sensory signs. *Can sometimes be associated with central neuropathological pain. ^‡^Can sometimes be associated with peripheral neuropathological pain^9^ [Color figure can be viewed at wileyonlinelibrary.com]

The use of only anticonvulsants, antidepressants, and other drugs is not ideal for the treatment of NP; the pain relief achieved is minimal and there are many side effects.^51^ For patients suffering from pain for a long time, extensive research has shown that SCS is one of the effective and safe methods to treat NP. Tanei et al.^52^ found that 7 days of SCS treatment led to pain relief in NP patients, and for most patients, this was maintained for 1 year and beyond. Chakravarthy et al.^53^ also found that patients who could not work due to pain were more likely to return to work after SCS treatment. SCS was found to be safe, and most complications could be resolved by implantation of SCS devices, which were rarely life‐threatening.^54^ SCS has clinical applications in all kinds of NP, such as NP after herpes, neuralgia pain, trigeminal neuralgia, root disease, diabetes, neuropathy, HIV infection, leprosy, amputation, peripheral injury pain, nerve injury and stroke (in the form of central poststroke pain), and so on, and has yielded good curative effects (Figure 4).

### 4.1 PHN

The incidence of PHN is 9%–34%, and is positively correlated with age. Many patients show poor treatment response or unbearable side effects, so minimally invasive analgesia is sometimes required. Some prospective, randomized‐controlled trials have concluded that SCS is effective in alleviating PHN.^55^ KurKolinsky et al. found that some patients who received permanent SCS implantation experienced significant long‐term pain relief after short‐term SCS treatment,^56^ and Scowcroft et al. also found that short‐term SCS induced pain relief in PHN patients and to decreased anxiety and depression, and improved sleep quality after treatment.^57^ Studies also shown that treating ZAP with DRG and PRF performed better than sustained epidural blockade for ZAP. This method of nutriregulation may be an effective option for reducing the progression of neurological disease caused by persistent pain signal transmission after acute herpes zoster.^58^

### 4.2 Pudendal neuralgia

PN is an NP caused by inflammation, compression, or perineal nerve compression; the main treatment methods of PN include physical therapy, drug analgesia, nerve block, vaginal nerve decompression, and so forth. Buffenoir et al. found that patients with refractory PN who received spinal cone SCS experienced reduced maximum pain, average pain, and complications. This indicates that the spinal cord cone is a safe and effective novel therapeutic target of PN.^59^ PN is the most chronic and disabling form of perineal pain, which is poorly treated by decompression surgery; the use of a 16‐contact guide SCS may be useful for patients with refractory PN who cannot undergo decompression surgery as an effective and feasible treatment.^60^

### 4.3 FBSS

FBSS is the leading cause of chronic NP, affecting 40% of patients undergoing lumbosacral surgery for back pain.^61^ In an analysis of FBSS patients who underwent SCS or spinal resurgery between 2000 and 2009, Lad et al.^61^ found that the complication rate of SCS was significantly lower than that of spinal resurgery within 90 days; thus, incorporation of SCS into the median treatment of chronic pain in FBSS patients should be considered. In a detailed and comprehensive review of the literature, Cameron^62^ found that in 747 patients affected by FBSS and treated with SCS, the overall treatment success rate was 62%. Also, Esin et al., by including three groups, found that in patients with FBSS, MTZ and TZS should be treated taking into account SCS. The study showed the efficacy of APAH, fluvoxamine, and milnacipran along with SCS.^63^ Do et al.,^64^ by analyzing 208 FBSS patients who had been treated for more than 3 years, found that although tonic and HF10 stimulation did not differ in terms of reducing pain, further research is needed to detect differences between SCS and other waveforms. De Groote et al.^65^ studied 22 FBSS patients who received an magnetic resonance imaging protocol both before and for 3 months after surgery; the first finding that SCS is able to induce changes in gray matter and white matter volume indicates that there is an altered reversibility in the brain after treatment of chronic pain with SCS.

### 4.4 Phantom limb pain

Phantom limb pain is a chronic NP that is experienced by 50%–80% of amputees and the analgesics do not adequately control chronic pain caused by phantom limb pain. When pain relief through medication alone is not successful, surgical options have been proven to be effective.^66^ Using SCS, Raut et al.^66^ found that patients with phantom limb pain experienced better pain relief, and their VAS decreased from (8/10) to (2/10), suggesting that SCS can be used to treat phantom limb pain. In addition, North and Sharan^67^ reported the case of a 61‐year‐old man, in whom it was found that good pain relief was achieved, and the VAS score decreased significantly, indicating that SCS can be used to treat phantom limb pain. In brief, SCS is an important mode of treatment for phantom limb pain because it is effective in reducing pain in patients with phantom limb pain, improving their quality of life, and reducing the use of analgesics.

### 4.5 Painful diabetic neuropathy (PDN)

Diabetes is a common chronic disease, in which PDN is considered to be the most disabling and medically costly complication of diabetes.^68^ It may be present in one third of diabetic patients. It is also a heterogeneous group of disorders with an extremely complex pathophysiology and affects both the somatic and autonomic components of the nervous system. De Vos et al.^69^ found that after SCS treatment, patients with PDN experienced significant pain relief and had improved quality of life than before. This result was also confirmed by Beek et al.,^70^ who found that PDN patients treated with SCS had less pain and better quality of life than those treated with drugs.

### 4.6 Cephalic and facial neuralgia

**Figure 5:**
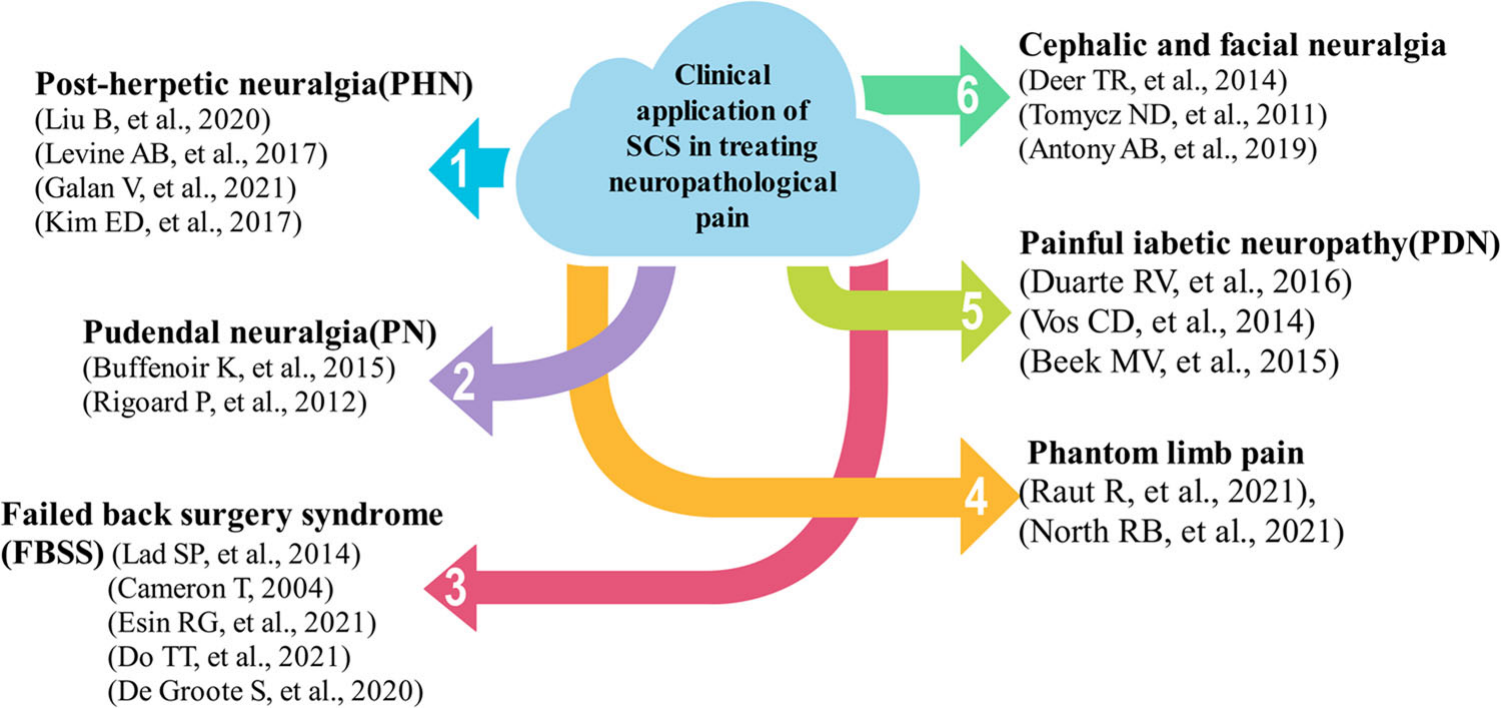
Clinical application of spinal cord stimulation (SCS) in treating neuropathological pain. This figure is a summary of the clinical application of SCS in the treatment of neurological pain [Color figure can be viewed at wileyonlinelibrary.com]

The higher cervical segment and the cervical–medullary junction (CMJ) region may be a new target for SCS therapy, and can be used to treat pain in the head and face.^71^ Chivukula et al. found that after receiving neck or CMJ zone SCS treatment, patients with head and facial neuralgia experienced pain relief.^72^ In the majority of patients, it was found that SCS treatment improved their quality of life and there was willingness to undergo surgery again for pain relief. Tomika et al. also confirmed this result. Velasquez et al. found that high cervical SCS can lead to pain relief in patients with trigeminal neuralgia. Although there are few studies and clinical applications of SCS therapy in the high cervical segment and CMJ region, it has been proved to be an effective target for the treatment of head and face pain, so it can be used as a reasonable and effective choice in clinical treatment. With the advancements of technology, neuromodulators may be promising in the treatment of intractable headaches and facial pain. Although more randomized‐controlled trials are needed to demonstrate efficacy and feasibility, the current literature sufficiently supports the view that neuromodulators are effective for the treatment of intractable craniofacial pain^73^ (Figure 5).

## 5 EFFICACY OF SCS IN TREATING NEUROPATHOLOGICAL PAIN

SCS is a recognized treatment for chronic NP. In recent years, increasingly more neurogenic chronic pain syndromes have been treated by SCS.^74^ Tanei et al.^52^ found that the NP patients could be relieved after 7 days of SCS treatment, and the pain relief effect of most patients lasted for more than 1 year. SCS has been found in related studies to improve pain and cramps and reduced use of painkillers. This also indicates the safety of SCS in SCI and suggests potential pain relief benefits.^75^ Eldabe et al.^54^ found that SCS is safe, and most of the complications could be treated by the removal of SCS devices, rarely endangering the patient's life. SCS is a useful treatment for neuropathological pain.

## 6 TECHNOLOGICAL ADVANCES IN SCS

### 6.1 HF‐SCS

**Figure 6:**
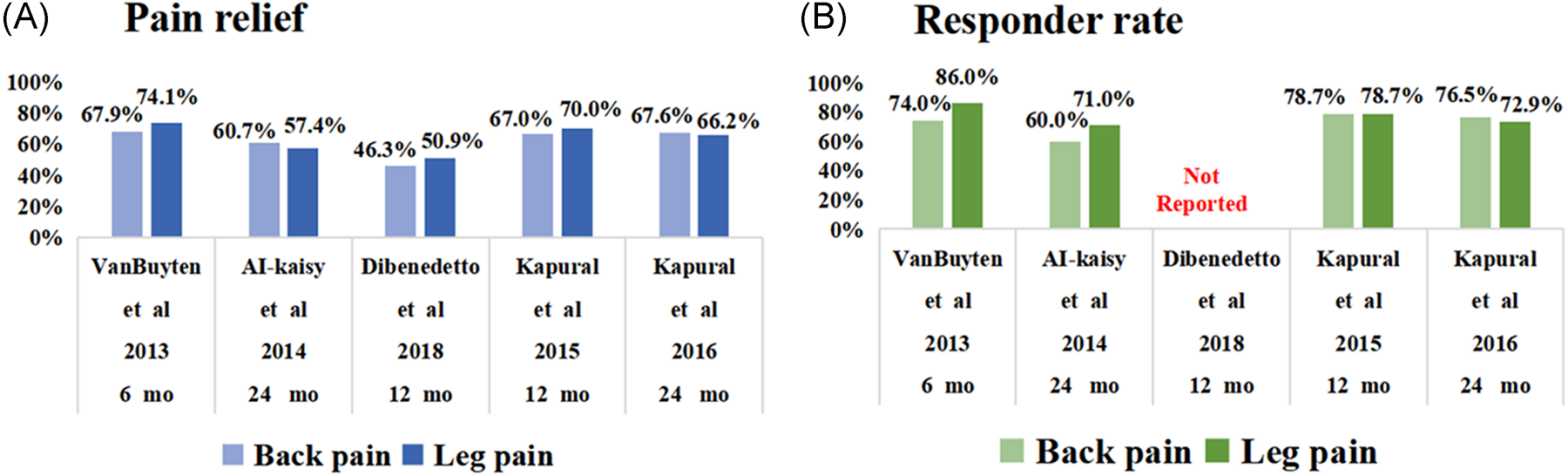
(A) Mean pain relief. (B) Responder rate.  10 kHz spinal cord stimulation benefits for patients with low back and leg pain [Color figure can be viewed at wileyonlinelibrary.com]

SCS is a recognized treatment. New 10 kHz high‐frequency therapy (HF10 therapy) outperformed conventional low‐frequency SCS in the treatment of chronic lumbago in a prospective randomized‐controlled trial.^76^ Vajramani^76^  used 10 kHz high‐frequency therapy (HF10 therapy) and the percutaneous SCS implant technique and found that the patient reported 90% pain reduction at follow‐up, indicating that thoracic 10 kHz high‐frequency SCS is an effective modality in managing chronic neuropathological pain. By assessing the efficacy of HF10 cSCS for chronic neck and/or upper limb pain, El Majdoub et al.^77^ found that patients had lower pain scores and reduced pain, demonstrating that HF10 cSCS is a treatment option that can lead to pain relief without causing paresthesia. By searching MEDLINE and EMBASE databases, it was concluded that pain scores improved after HF‐SCS treatment, indicating that HF‐SCS was useful for pain control in patients with BISphenol A.^78^ Salmon^79^ found a decrease in all relevant indicators after using the hf10‐SCS guide treatment with the cervical and thoracic vertebrae in 45 patients with NP in the upper and lower limbs, suggesting that this approach can achieve both cost reduction and improved clinical outcomes. These results suggest that HF10‐SCS may be a viable alternative for patients with refractory pain who cannot receive traditional SCS therapy (Figure 6).

### 6.2 Burst SCS

Recently, a novel mode of stimulation has been proposed as an excellent alternative for the treatment of persistent central nervous pain, namely, sudden stimulation. Yoon and Kim^80^ used burst SCS and found that the efficacy of burst SCS in central neuropathological pain is desirable. We all know that new advancements in SCS treatment can provide effective pain relief in patients with chronic pain. Lee et al.,^5^ by inserting a pulse mode spinal cord stimulator, observed improvements in patients' function and pain relief, indicating that a pulse mode spinal cord stimulator can be used safely and effectively in the treatment of patients with PCP. Burst SCS is a sensorless waveform that can be used to treat NP, but the mechanism may be distinguished from tonic SCS. Royds et al.,^81^ by implanting SCS, found significant changes in the expression of proteins associated with synaptic assembly and immune regulation, indicating that cerebrospinal fluid proteome changes are mainly associated with synaptic assembly and immune effectors. Explosive SCS can effectively lead to pain relief in FBSS patients and improve sleep quality and relieve depression.

### 6.3 DRGS

DRGS is an effective method to treat chronic pain such as lumbago, pelvic pain, and complex local pain syndrome. Chemotherapy‐induced neuropathy can be treated with DRGS.^82^ DRGS can reduce the excitability of neurons, act directly, and repair pathological neurons in the dorsal root ganglia, achieving an analgesic effect.^83^ Hunter and Yang^84^ found that in patients with refractory chronic pelvic pain who had undergone numerous invasive treatments (including SCS), but showed poor outcomes, pelvic pain improved significantly after DRGS treatment at bilateral L1, S2 levels and led to reduced opioid dosage. Neuromodulation is an important means to achieve relief of NP. DRG stimulation before primary sensory neurons enter the spinal canal is a high level of tumor‐specific skin treatment that is superior to traditional SCS. A retrospective review of a single permanent DRG stimulus for more than three years by Verrills et al.^85^ found significant and sustained relief of pain, indicating the need for future prospective studies of DRG stimulation below the sensory abnormality threshold.^85^ Seventy‐five patients (41 DRG, 34 SCS) were scanned and digitally compared with sufficient‐quality maps, indicating that the average frequency of DRG stimulation‐produced paresthesia was lower and less location dependent, showing some potential in the treatment of NP.^86^ The new SCS electrical stimulation mode yields good therapeutic effects in NP treatment, especially traditional SCS treatments.

## 7 CONCLUSIONS AND PROSPECTS

### 7.1 Efficacy of spinal cord electrical stimulation

Chronic ache is the most common manifestation of neuropathological pain, and it markedly affects the quality of life of patients. Therefore, studies of the occurrence, development, and maintenance mechanism of neuropathological pain should be carried out to identify treatments that can help relieve and control pain more effectively, so a large number of neuropathological pain animal experimental models were developed. The main purpose of these studies is to find the pain occurrence mechanism. SCS therapy has been widely used in the treatment of a variety of diseases and it can lead to significant pain relief in NP patients and improve their quality of life.

### 7.2 Further elucidation of the mechanism of action of SCS

SCS can be used to treat a variety of diseases in a multidisciplinary and specialized field. SCS treatment can lead to significant pain reduction in NP patients and improve their quality of life. The emergence of more new targets and electrical stimulation modes will provide more ideas and options for SCS treatment of NP. Studies should be carried out to further clarify the mechanism of SCS, and new modes of electric stimulation should be developed continuously. SCS can inhibit or reduce pain by blocking pain signal transmission, interfering with the pain pathway, activating the opioid pathway, stimulating the locus coeruleus system, and regulating GABA energy.

### 7.3 Prospects

**Figure 7:**
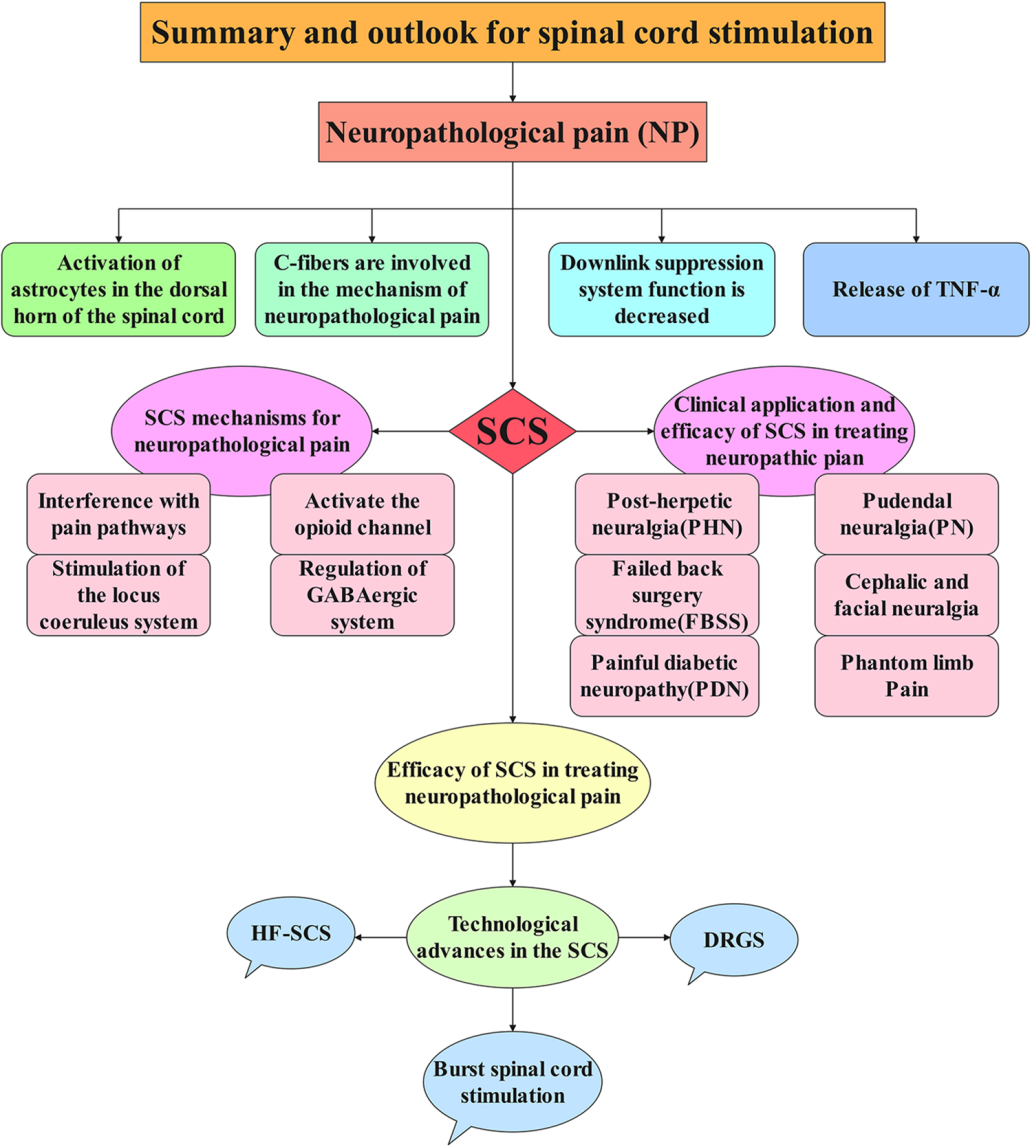
Summary and outlook for spinal cord stimulation. This figure is a summary of the full‐text content. DRGS, dorsal root ganglion stimulation; HF‐SCS, high‐frequency SCS; SCS, spinal cord stimulation; TNF, tumor necrosis factor [Color figure can be viewed at wileyonlinelibrary.com]

The cost of electrical SCS is higher than other treatments such as drugs, and the prevalence rate is low. It may be impossible to use SCS to treat the corresponding diseases in a few cases, and even using SCS treatment is also difficult to reduce the pain. Therefore, it is very important to continue to develop new electric stimulation models and look for new stimulation targets, and the practicality of costs should also be within the scope of our study to achieve more development and clinical applications (Figure 7).

## CONFLICT OF INTERESTS

The authors declare no conflict of interests.

## ETHICS STATEMENT

Not applicable.

## AUTHOR CONTRIBUTIONS

Shun‐Lian Li contributed the central idea and wrote the initial draft of the paper. Jing Li, Hui‐Chan Xu, Yu‐Cong Liu, and Ting‐Ting Yang contributed to refining the ideas and carrying out additional analyses. Hao Yuan reviewed and edited this paper.
